# Understanding the etiology of diarrheal illness in Cambodia in a case-control study from 2020 to 2023

**DOI:** 10.1186/s13099-025-00709-0

**Published:** 2025-05-22

**Authors:** Paksathorn Kietsiri, Siriporn Sornsakrin, Samon Nou, Wilawan Oransathid, Dutsadee Peerapongpaisarn, Wirote Oransathid, Panida Nobthai, Patcharawalai Wassanarungroj, Siriphan Gonwong, Pimmada Sakpaisal, Nuanpan Khemnu, Somethy Sok, Sokh Vannara, Chiek Sivhour, Sidonn Krang, Ly Sovann, Em Sovannarith, Woradee Lurchachaiwong, Sidhartha Chaudhury, Nattaya Ruamsap, Paphavee Lertsethtakarn

**Affiliations:** 1 Walter Reed Army Institute of Research– Armed Forces Research Institute of Medical Sciences, Bangkok, Thailand; 2 Walter Reed Army Institute of Research– Armed Forces Research Institute of Medical Sciences, Phnom Penh, Cambodia; 3 Military Region 5 Hospital, Battambang, Cambodia; 4 Battambang Referral Hospital, Battambang, Cambodia; 5 Communicable Diseases Control Department, Ministry of Health, Phnom Penh, Cambodia

**Keywords:** Acute diarrhea, Antibiotic resistance, ETEC, *Shigella*, Rotavirus

## Abstract

Diarrheal infection remains a major public health problem in low and middle-income countries (LMICs). Prevention and control of diarrheal diseases are considered a global health priority. This case-control study aims to describe the prevalence of diarrhea etiologic agents and antimicrobial resistance in bacterial enteropathogens for acute diarrhea among children, adult civilians, and military personnel in Cambodia, detecting over 20 bacterial species, viruses, and parasites. A total of 918 subjects with acute diarrhea (cases), 791 aged-matched subjects without diarrhea (controls), and 675 follow-up cases were enrolled from five hospitals in Battambang and Oddor Meanchey provinces from 2020 to 2023. Pathogens were identified from collected stool samples via bacteriology, molecular techniques, immunoassays, and microscopy. Bacterial isolates were tested for antibiotic resistance patterns. From enrolled diarrhea cases, 533 stool samples (58%) were positive for enteric pathogens, compared to 389 samples (49%) in controls, underscoring the high carriage rate of enteric pathogens in this population as well as the difficulties in establishing the etiology of diarrhea cases. The most common enteric pathogens in cases were enteric bacteria with *Aeromonas* (15%), followed by *Plesiomonas* (12%), and enteroaggregative *E. coli* (EAEC) (10%). *Shigella* (*p* < 0.05), enterotoxigenic *E. coli* with heat-stable toxins (ETEC-ST) (*p* < 0.01), and *Plesiomonas* (*p* < 0.01) had a statistically significant association with acute diarrhea cases. Rotavirus was the most common virus found (51% of cases with virus), followed by norovirus (19%), and sapovirus (16%). In terms of antimicrobial resistance, 84% of *Shigella* isolates were highly resistant to trimethoprim/sulfamethoxazole (SXT), almost 80% of *Campylobacter jejuni* isolates were resistant to ciprofloxacin (82%) and nalidixic acid (85%). Over 50% of ETEC, *Shigella*, and EAEC isolates were resistant to ceftriaxone, ciprofloxacin, and SXT, respectively. Overall, our study highlights the high endemicity of enteric bacterial pathogens and the significant carriage rates of these pathogens even in individuals without overt symptoms. Although the overall antimicrobial resistance was moderate, prevalent isolates harbor a significant resistance to the first-line of treatment. This highlights the importance of ongoing diarrhea etiology and antimicrobial resistance (AMR) surveillance efforts to guide the development and implementation of an effective AMR management program in diarrheal infections.

**Supplementary Information:**

The online version contains supplementary material available at 10.1186/s13099-025-00709-0.

## Introduction

Diarrheal infection is a serious public health problem in low and middle-income countries (LMICs) and is the second leading cause of death in children under five years old [1]. Globally, there are nearly 1.7 billion cases among children and adults reported annually [2, 3]. Over 440,000 children under the age of five die of complications from diarrheal infections annually [2]. Diarrheal infection can also lead to malnutrition in children under five years old [2]. The USA and Germany have reported that 0.95 episodes of acute gastrointestinal illness occur per person per year in adults [4, 5], and more than 5,000 adults die annually [3]. In Cambodia, diarrheal infection impacts all age groups [6]. Consumption of contaminated food or drinking water, poor hygiene, or extreme weather events (such as floods, droughts, and typhoons) are some of the leading causes of diarrheal infection in Cambodia [2, 7]. Socioeconomic factors were also reported to be linked to diarrheal infection [6].

The most recent report of diarrhea etiology in Cambodia was a multi-year cohort study conducted in 2012–2018, which identified parasites, mainly *Blastocystis hominis*, in over half of all cases whereas 38% of samples were positive for bacteria [6]. Viruses were identified in only 3% of collected samples [6]. Although antibiotic treatment is not required in most diarrheal cases, the overuse of antibiotics to treat other infections may cause antimicrobial resistance (AMR) development among bacterial species [8, 9]. It may also alter the gut microbiota in ways that can increase susceptibility to intestinal pathogens [10]. Southeast Asia has been identified as an area of great importance in the development and spread of AMR among humans [11]. There are few articles that report the AMR rates among enteric pathogen isolates from human stool samples in Cambodia, however, an increasing AMR trend among enteric pathogens has been reported [12].

Studies on AMR in enteric pathogens in Southeast Asia have typically focused on two distinct populations: civilians (usually children), and Western travelers and military personnel. Studies on civilians have reported the emergence of antibiotic drug resistance since the 1990s. Several studies have reported that the enterotoxogenic *E. coli* (ETEC) show resistance to trimethoprim/sulfamethoxazole (SXT), ampicillin (AMP), chloramphenicol, and tetracycline [13, 14]. Others have found that *Campylobacter* spp. show high resistance to fluoroquinolones such as ciprofloxacin (CIP) [14, 15] and moderate resistance to macrolides such as azithromycin (AZM), and that *Shigella* spp. show high resistance to fluoroquinolones [12]. Studies on Western travelers and military personnel have tended to focus on *Campylobacter* spp. and show high resistance to fluoroquinolones and tetracyclines, and moderate resistance to SXT [16–18]. Overall, these findings over twenty years show increasing trends of drug resistance for several key classes of drugs that are commonly prescribed for treatment of acute disease and self-administration for Western travelers, including SXT (sold under trade names of Bactrim, Cotrim, and Septra, among others), CIP, and AZM (sold under trade names Zithromax and Azasite). Ongoing surveillance of these antimicrobial resistance patterns is critical to ensure that current prophylactic and treatment guidelines remain appropriate for the bacterial enteropathogens prevalent in Southeast Asia.

In this case-control study, we determine the prevalence of bacterial, parasitic, and viral etiological agents for acute diarrhea as well as the antimicrobial resistance patterns for bacterial enteropathogens in acute diarrhea cases for children, adults, and military personnel in Battambang and Oddar Meanchey provinces, Cambodia. We assess the symptoms of acute diarrhea and the medical interventions used to treat it. We analyze the association between the clinical presentation of acute diarrhea and its specific symptoms and conduct follow-up assessments to characterize the resolution of the diarrhea symptoms.

## Materials and methods

### Ethics statement

The human use protocol was reviewed and approved by the Cambodia National Ethics Committee for Health Research (NECHR) and the Walter Reed Army Institute of Research (WRAIR) Institutional Review Board. Subjects or subjects’ guardians provided written informed consent before participation.

### Study site and enrollment

The surveillance was conducted from April 2020-August 2023 at Battambang Referral Hospital, Military Regional 5 Hospital, and SvayPor Health Center in Battambang province, Anlong Veng Referral Hospital and Oddar Meanchey hospital in Oddar Meanchey province in Cambodia and included both civilian and military populations.

Patients in the inpatient and outpatient departments who sought medical care for acute diarrhea (case) and other non-diarrheal conditions (control) were invited to participate and enroll in the study. Acute diarrhea cases were defined as having three or more loose or liquid stools per day with a reported change in the patient’s baseline bowel movements. Age-matched control was enrolled for each diarrheal case by stratifying into age groups: 3 months-1 year; 2–5 years; 6–17 years; and 18–60 years of civilians and active duty military personnel. Follow up cases were invited to follow up at the clinic/hospital at 7–21 days after enrollment regardless of whether the participant had recovered or was still experiencing diarrhea at the time of the follow up. The stool samples were collected from cases, controls, and follow up cases after informed consent had been obtained.

### Pathogen detection, isolation, and identification

The stool samples were collected in triple packaging system (stool cups, specimen bags, and styrofoam boxes) with ice packs and transported to the microbiology laboratory within 4 h to ensure that all culturable pathogens were alive for processing. Direct stool microscopic examination and a formalin-ethyl acetate sedimentation concentration technique were performed for the detection of ova and parasites [14]. Bacterial isolation and identification were performed as follows. Stool samples were resuspended and inoculated into a range of selective media and enrichment broths: MacConkey, Hektoen, thiosulfate citrate bile salts sucrose, modified semisolid Rappaport Vassiliadis, modified charcoal cefoperazone deoxycholate agar, buffered peptone water, alkali peptone water, and Preston selective enrichment broth in order to culture *Aeromonas*, *Arcobacter*, *Campylobacter*, *Escherichia coli*, *Plesiomonas*, *Salmonella*, *Shigella*, *Vibrio* spp., and *Yersinia enterocolitica* [19]. Selective plates were used to examine the presence of colonies resembling *Shigella*, *Vibrio*, *Salmonella*, *E. coli*, *Aeromonas*, *Plesiomonas*, *Yersinia*, *Campylobacter*, and *Arcobacter.* To determine *Shigella* and *Vibrio* isolates subtype, Denka-Seiken antisera was used (Denka-Seiken, Tokyo, Japan). *Salmonella* isolates were serogrouped using antisera (S&A Reagents Lab, Bangkok, Thailand) to determine the subgrouping. *Campylobacter* and *Arcobacter* speciation were determined by phenotypic biochemical properties [20]. All other bacterial isolates identified were confirmed by biochemical testing [21]. Diarrheagenic *E. coli* (DEC) were tested by a multiplex Polymerase Chain Reaction (PCR) to determine the pathotypes: Enteroaggregative *E. coli* (EAEC), Enteroinvasive *E. coli* (EIEC), Enteropathogenic *E. coli* (EPEC), ETEC, and Shiga toxin-producing *E. coli* (STEC) (Supplement Table 1) [22, 23]. Enteric parasites and viruses were identified using commercial ELISA kits for *Giardia* and *Cryptosporidium* (TECHLAB^®^, Blacksburg, VA, USA), adenovirus, astrovirus, and rotavirus (RIDASCREEN^®^, R-Biopharm AG, Darmstadt, Germany) and by TaqMan^®^ probe based reverse transcription real-time PCR to detect norovirus GI, norovirus GII, and sapovirus. Primer and probe sequences and assay details are available in listed references [24, 25].

### Antimicrobial sensitivity testing

All bacterial pathogen isolates except *Campylobacter* and *Arcobacter* were tested for antimicrobial susceptibility to six antibiotics (Becton Dickinson and Company, MD, USA) including ampicillin (AMP), azithromycin (AZM), ceftriaxone (CRO), ciprofloxacin (CIP), nalidixic acid (NAL), and trimethoprim/sulfamethoxazole (SXT) by disc diffusion method according to Clinical and Laboratory Standards Institute (CLSI, 2020) guidelines and as previously described [26, 27]. *Campylobacter* and *Arcobacter* isolates were tested for antimicrobial susceptibility to erythromycin (ERY), AZM, CIP, and NAL by Etest (Liofilchem, Roseto degli Abruzzi, Italy) according to the National Antimicrobial Resistance Monitoring System for Enteric Bacteria (NARMS) [28]. Breakpoints for *Campylobacter* spp. by NARMS were used for the interpretation of *Arcobacter* spp. susceptibility results as they belong in the same Family. Multidrug resistance (MDR) was defined as resistance to at least one antimicrobial agent in three or more antimicrobial drug classes [29]. Supplement Table 2 provides the number of isolates tested on different antibiotics.

### Statistical methods

Statistical analysis was performed using the R statistical software package version 4.4.1 (R Development Core Team). Carriage rate was defined as the proportion of individuals in the study that were positive for a given pathogen. To determine the pathogenicity of carriage, we used logistic regression to calculate an adjusted Odds Ratio (aOR) comparing the detection of a particular pathogen in cases vs. controls, adjusting for age group and sex. Four age groups were used: infants (3 months-1 year), young children (2–5 years), children and adolescents (6–17 years), and adults (18–60 years). We calculated symptomicity comparing the detection of a particular pathogen in acute diarrhea cases presenting with a particular symptom compared to cases where that symptom was not present, adjusting for age-group and sex. We also calculated the association of pathogen with hospitalization, accounting for age group and sex. P-values are reported from the logistic regression reflecting the likelihood of rejecting the null hypothesis that the aOR is equal to 1.0. Logistic regression was only carried out for pathogens for which there were at least 10 positive cases.

## Results

### Demographics

A total of 1,709 participants (918 cases and 791 controls) were enrolled in the study, with follow-ups conducted for 675 cases. The demographics of cases and controls, and clinical information of cases and follow up cases are shown in Table 1. A breakdown of enrollment by hospital site is provided in Supplement Table 3. Overall, 1,220 subjects were enrolled in inpatient departments (IPD) and 489 subjects were enrolled in outpatient departments (OPD). Approximately 11% of subjects were infants (3 months-1 year), 26% of subjects were young children (2–5 years), 8% of subjects were children and adolescents (6–17 years), and 55% of subjects were adults (18–60 years). Overall, 45% of study subjects were children, 52% were adult civilians, and 3% were adult active-duty military. Approximately 46% of subjects 5 years old and younger were still breastfeeding.

**Table 1:** Study demographics

Enrollees	Cases *n* (%)				Controls *n* (%)			
	*N* = 918				*N* = 791			
	IPD		OPD		IPD		OPD	
	*N* = 711		*N* = 207		*N* = 509		*N* = 282	
**Age group**	**N**	**%**	**N**	**%**	**N**	**%**	**N**	**%**
**Mean**,** SD**	25.9 ± 20.89		14.7 ± 19.1		24.6 ± 20.9		24.7 ± 21.1	
3mths − 1 yr	70	10%	62	30%	30	6%	38	13%
2–5 yrs	168	24%	61	29%	145	28%	63	22%
6–17 yrs	51	7%	10	5%	54	11%	24	9%
18–60 yrs	422	59%	74	36%	280	55%	157	56%
**Gender**								
Male	406	57%	114	55%	284	56%	158	56%
Female	305	43%	93	45%	225	44%	124	44%
**Status**								
Children	289	41%	133	64%	229	45%	125	44%
Adult civilians	401	56%	68	33%	276	54%	144	51%
Active duty military personnel	21	3%	6	3%	4	1%	13	5%
**Breast Feeding**								
Still breastfeeding (≤ 5yrs only)	103	43%	84	68%	52	30%	54	53%

### Clinical presentation and outcomes

The clinical presentation of acute diarrhea symptoms is shown in Table 2 for both IPD and OPD subjects. In both groups, the median duration of acute diarrhea prior to enrollment was approximately 2.5 days. In terms of stool consistency, the most commonly reported characteristic was loose stool (61% of all cases) and watery stool (45% of all cases). Among subjects enrolled in IPD, there was a 21% rate of mucous and 5% rate of bloody stool. Abdominal pain was the most widely reported symptom at 76% of all cases. Other common symptoms included fatigue, nausea, anorexia, vomiting, and fever. In general, subjects in the IPD had much higher rates of common symptoms (55–75%) than OPD subjects (17–37%), suggesting a more severe clinical presentation in IPD.

**Table 2:** Clinical presentation of acute diarrhea cases

Clinical information	IPD		OPD	
	*N* = 711		*N* = 207	
	*N*	%	*N*	%
Duration (days) (Mean ± SD)	2.7 ± 1.3		2.5 ± 1.1	
**Stool characteristics**				
Loose	419	59%	139	67%
Watery	350	49%	67	32%
Mucous	146	21%	10	5%
Bloody	32	5%	1	0%
**Symptoms**				
Abdominal Pain	539	76%	151	73%
Fatigue	533	75%	74	36%
Nausea	499	70%	77	37%
Anorexia	455	64%	51	25%
Vomiting	450	63%	35	17%
Fever	394	55%	57	28%

We completed follow-ups for 675 participants in order to record the type of treatment given, whether or not their diarrhea symptoms were resolved, how long the symptoms took to resolve, and the length of their hospital stay (Table 3). We found that in the follow up cases, 75% of subjects were hospitalized and the average length of the hospital stay was 3.9 days. In the IPD, subjects were generally given IV fluids (72%) and antibiotics (91%), but were also sometimes given oral rehydration (ORS) (38%) and Zinc (21%). In OPD subjects, antibiotics were prescribed more sparingly (34%), and treatment primarily consisted of ORS (68%) and Zinc (60%). Overall, among IPD subjects, 93% reported that the diarrhea was resolved with an average time to resolution of 3.6 days. For OPD subjects, 88% reported resolution of their symptoms with an average duration of 3.0 days.

In terms of antibiotics, the following were prescribed most frequently: SXT (37%), metronidazole (15%), CIP (11%), amoxicillin (9%), cefixime (7%), AZM (4%), and CRO (2%). The results showed that 44% of antibiotic-treated cases were, in fact, infected by bacteria while the remaining antibiotic-treated cases were infected by viruses or parasites or had no pathogens identified. Of those who were infected by bacteria initially, 12% were not given any antibiotic treatment at enrollment (data not shown).

**Table 3:** Treatment and outcome at follow-up

Clinical information	Cases			
	*N* = 675			
	IPD		OPD	
	*N* = 504		*N* = 171	
	*N*	%	*N*	%
Hospitalization duration (days)	3.9 ± 2.0			
**Treatment type given**				
IV fluid	364	72%	2	1%
Antibiotic*	461	91%	58	34%
Trimethoprim/Sulfamethoxazole	211	42%	37	22%
Metronidazole	91	18%	9	5%
Ciprofloxacin	66	13%	7	4%
Amoxicillin	54	11%	4	2%
Cefixime	44	9%	0	0%
Azithromycin	27	5%	0	0%
Ceftriaxone	15	3%	0	0%
Other antibiotics	10	2%	1	1%
ORS	193	38%	117	68%
Zinc	108	21%	103	60%
**Outcome**				
Diarrhea resolved	471	93%	151	88%
Days to resolution	3.6 ± 1.7		3.0 ± 1.8	

*One subject may have received multiple antibiotics

### Pathogen identification

**Fig. 1:**
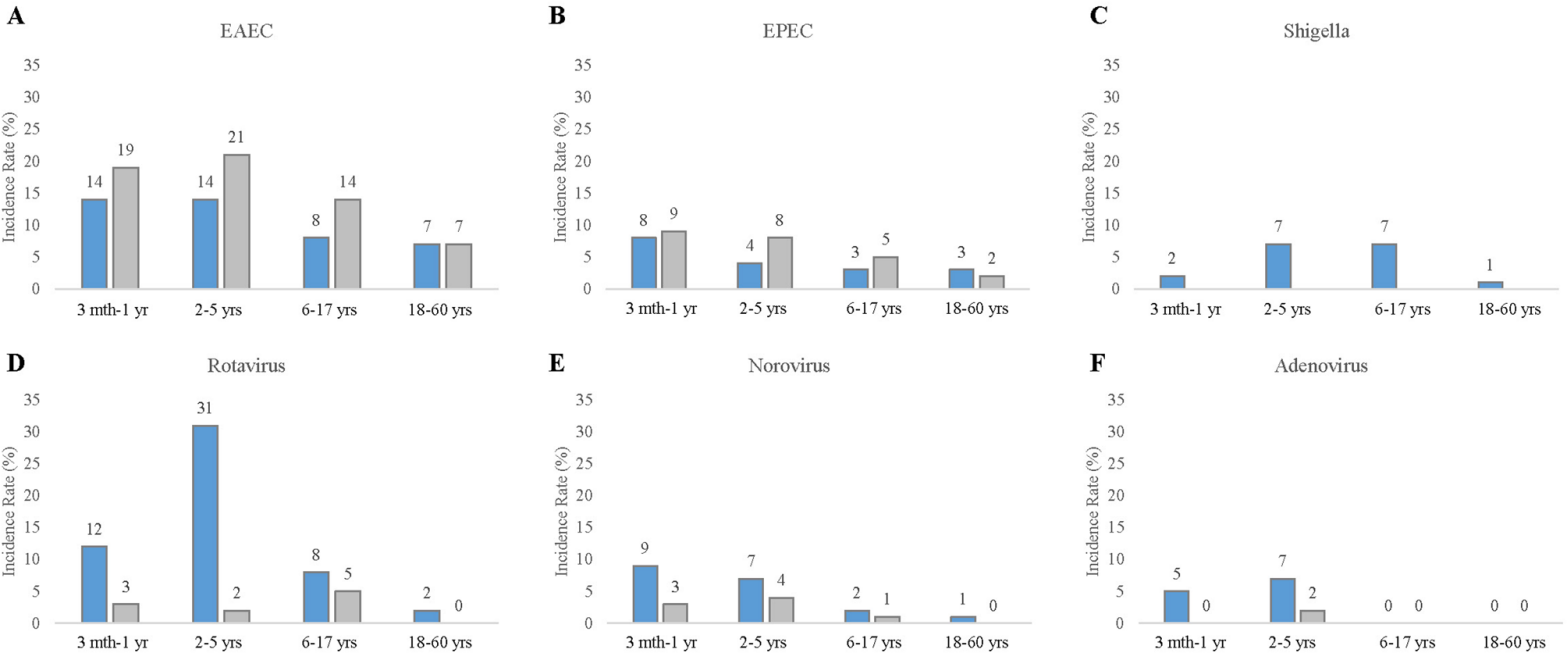
Incidence rate of enteric pathogens in cases and controls. Incidence rate is shown for cases (blue) and controls (gray) for several key bacterial and viral enteric pathogens in four age groups: infants (3 months-1 year), young children (2–5 years), children and adolescents (6–17 years), and adults (18–60 years)

The most common pathogen identified in positive cases was bacterial (383 of 918 cases, 42%), followed by viruses (180 cases, 20%) and parasitic pathogens (67 cases, 7%). *Aeromonas* was the most prevalent (138 cases, 15%), followed by *Plesiomonas* (110 cases, 12%), and DEC, specifically EAEC (92 cases, 10%). The most prevalent enteric virus and parasite in cases were rotavirus (99 cases, 11%) and *Giardia* (47 cases, 5%), respectively. A total of 533 case samples were positive for enteric pathogens with 202 samples containing multiple pathogens. The overall co-infection rate of bacteria and virus was relatively low at 6% (Supplement Table 4). A significant number of bacterial and parasite-positive samples were present at a comparable percentage in both cases and controls, notable were *Aeromonas*, *Plesiomonas*, *Salmonella*, EPEC, *Campylobacter*, *Arcobacter*, and *Giardia* (Table 4). We found that overall, there was a high carriage rate for bacterial pathogens in both the case and control subjects. For DEC such as EAEC and EPEC (Fig. 1A and B), we found that infants and children showed particularly high aggregate carriage rates of 23–31%, compared to adults (9–11%).

For bacterial pathogens, a logistic regression analysis to assess association with case vs. control, revealed some associations with bacterial pathogens and acute diarrhea. For *Shigella*, we observed that while its prevalence was relatively low, it was highly associated with acute diarrhea cases (*p* < 0.01) with an aOR of 23.4, mainly among children and adolescents. Of the 28 isolates of *Shigella* found in this study, 54% were *S. flexneri* and 39% were *S. sonnei*. For *Plesiomonas*, we found a weak association with acute diarrhea cases (*p* < 0.05) with an aOR of 1.4, mainly in the children and adolescents group (6–17 years) and adults. For all bacterial pathogens, we carried out a further analysis using pathogen subtypes to determine whether a particular subtype was associated with acute diarrhea cases. Results for the subtypes of each pathogen are listed in Supplemental Table 5. We found that although ETEC did not show a statistically significant association with diarrhea, ETEC with heat-stable toxin (ETEC-ST) showed a significant association (*p* < 0.05) with an aOR of 4.4. For *Campylobacter*, we found that both *C. coli* and *C. jejuni* showed relatively high aORs of 2.1 and 1.5, respectively, but it was not statistically significant, likely due to the small number of cases in Table 4.

**Table 4:** Pathogen identification in cases and controls

Pathogens	Infants (3 months-1 year)				Young children (2–5 years)				Adolescent (6–17 years)				Adult (18–60 years)				aOR†(95% C.I.)	
	Case	%	Control	%	Case	%	Control	%	Case	%	Control	%	Case	%	Control	%		
BACTERIA																		
*Aeromonas*	16	12%	11	16%	44	19%	27	13%	5	8%	11	14%	75	15%	64	15%	1.1 (0.8, 1.4)	
Diarrheagenic *E. coli*	30	23%	21	31%	55	24%	64	31%	7	11%	17	22%	56	11%	41	9%	0.8 (0.6, 1.0)	
EAEC	19	14%	13	19%	31	14%	43	21%	5	8%	10	13%	35	7%	32	7%	0.8 (0.5, 1.1)	
EPEC	10	8%	6	9%	10	3%	16	8%	2	3%	4	5%	13	3%	7	2%	0.9 (0.5, 1.4)	
ETEC	1	1%	2	3%	11	5%	3	1%	0	0%	3	4%	7	1%	1	0%	1.9 (0.9, 4.5)	
ETEC(LT)	1	1%	2	3%	3	1%	1	0%	0	0%	2	3%	1	0%	1	0%	0.7 (0.2, 2.3)	
ETEC(ST)	0	0%	0	0%	5	2%	1	1%	0	0%	0	0%	4	1%	0	0%	4.4 (1.4, 19.1)*	
ETEC (LT/ST)	0	0%	0	0%	3	1%	1	0%	0	0%	1	1%	2	0%	0	0%		
STEC	0	0%	0	0%	0	0%	1	0%	0	0%	0	0%	1	0%	1	0%		
EIEC	0	0%	0	0%	3	1%	1	0%	0	0%	0	0%	0	0%	0	0%		
*Plesiomonas*	9	7%	3	4%	17	7%	12	6%	11	18%	11	14%	70	14%	45	10%	1.4 (1.0, 1.9) *	
*Salmonella*	16	12%	5	7%	10	4%	28	13%	5	8%	5	6%	53	11%	45	10%	0.8 (0.6, 1.2)	
*Campylobacter*	8	6%	2	3%	11	5%	12	6%	2	3%	4	5%	8	2%	3	1%	1.2 (0.7, 2.1)	
*C. coli*	0	0%	0	0%	4	2%	3	1%	1	2%	1	1%	6	1%	1	0%	2.1 (0.8, 6.6)	
*C. jejuni*	8	6%	2	3%	7	3%	6	3%	1	2%	0	0%	2	0%	1	0%	1.5 (0.7, 3.6)	
*Shigella*	2	2%	0	0%	15	7%	1	0%	4	7%	0	0%	3	1%	0	0%	23.4 (5.0, 430)**	
*S. boydii*	0	0%	0	0%	1	0%	0	0%	0	0%	0	0%	1	0%	0	0%		
*S. flexneri*	0	0%	0	0%	9	4%	1	0%	3	5%	0	0%	2	0%	0	0%	145.6 (3.1, 284)**	
*S. sonnei*	2	2%	0	0%	5	2%	0	0%	1	2%	0	0%	0	0%	0	0%		
*Arcobacter*	0	0%	0	0%	3	1%	3	1%	1	2%	1	1%	7	1%	9	2%		
*Vibrio spp.*	0	0%	0	0%	0	0%	0	0%	1	2%	0	0%	1	0%	0	0%		
*Yersinia*	0	0%	0	0%	0	0%	0	0%	0	0%	0	0%	1	0%	0	0%		
PARASITES																		
*Giardia*	7	5%	2	3%	23	10%	30	14%	7	11%	13	17%	10	2%	8	2%	0.8 (0.5, 1.2)	
Hookworm	0	0%	0	0%	1	0%	0	0%	1	2%	0	0%	4	1%	3	1%		
*Opisthorchis*	0	0%	0	0%	0	0%	0	0%	0	0%	0	0%	5	1%	3	1%		
*Strongyloides*	0	0%	0	0%	0	0%	0	0%	0	0%	0	0%	3	1%	1	0%		
*Cryptosporidium*	2	2%	0	0%	1	0%	0	0%	0	0%	0	0%	2	0%	2	0%		
*E. histolytica*	0	0%	0	0%	2	1%	0	0%	0	0%	0	0%	1	0%	1	0%		
*Taenia*	0	0%	0	0%	0	0%	0	0%	0	0%	0	0%	1	0%	0	0%		
VIRUSES																		
Norovirus	12	9%	2	3%	17	7%	8	4%	1	2%	1	1%	7	1%	0	0%	2.8 (1.4, 5.8) **	
Sapovirus	3	2%	0	0%	23	10%	7	3%	0	0%	6	8%	4	1%	2	0%	1.9 (1.0, 3.7)*	
Adenovirus	5	5%	0	0%	13	6%	5	2%	0	0%	2	3%	3	1%	2	0%	2.1 (1.1, 4.7)*	
Astrovirus	2	2%	0	0%	4	2%	4	2%	0	0%	0	0%	0	0%	2	0% 33,888,880%		
Rotavirus	16	12%	2	3%	71	31%	5	2%	5	8%	4	5%	7	1%	2	0%	8.2 (4.6, 15.6)***	

aOR– adjusted odds ratio for cases vs. controls for each pathogen; C.I.– confidence interval

† logistic regression only run on pathogens for which there were at least 10 cases; p-values are noted: * *p* < 0.05, ** *p* < 0.01, *** *p* < 0.001

**Table 5:** Symptoms associated with enteric pathogens

Pathogens	Cases	Symptoms												Stool characteristics							
		Fever		Abdominal Pain		Nausea		Vomit		Fatigue		Anorexia		Watery		Loose		Mucous		Blood	
		*N*	%	*N*	%	*N*	%	*N*	%	*N*	%	*N*	%	*N*	%	*N*	%	*N*	%	*N*	%
All cases	918	451	49%	690	75%	576	63%	485	53%	607	66%	506	55%	417	45%	558	61%	156	17%	33	4%
BACTERIA																					
*Aeromonas*	140	65	46%	104	74%	81	58%	58	41%	96	69%	70	50%	70	50%	78	56%	32	23%	5	4%
Diarrheagenic *E col*i	148	67	45%	96	65%	78	53%	63	43%	81	55%	69	47%	51	34%	103	70%	21	14%	5	3%
EAEC	90	44	49%	58	64%	51	57%	39	43%	50	56%	44	49%	28	31%	63	70%	12	13%	2	2%
EPEC	35	13	37%	24	69%	16	46%	15	43%	21	60%	17	49%	10	29%	27	77%	4	11%	0	0%
ETEC	19	7	37%	12	63%	7	37%	5	26%	9	47%	7	37%	11	58%	10	53%	4	21%	2	11%
*Plesiomonas*	107	33	31%	90	84%	63	59%	47	44%	74	69%	51	48%	43	40%	65	61%	15	14%	4	4%
*Salmonella*	84	39	46%	69	82%	39	46%	29	35%	47	56%	41	49%	25	30%	56	67%	19	23%	2	2%
*Campylobacter*	29	12	41%	19	66%	15	52%	10	34%	11	38%	13	45%	12	41%	19	66%	4	14%	2	7%
*Shigella*	24	17	71%	15	63%	14	58%	12	50%	14	58%	13	54%	12	50%	9	38%	17	71%***	7	29%***
*Arcobacter*	11	5	45%	8	73%	8	73%	5	45%	8	73%	7	64%	7	64%	6	55%	2	18%	0	0%
PARASITES																					
*Giardia*	47	32	68%	27	57%	26	55%	24	51%	27	57%	23	49%	27	57%	25	53%	16	34%*	2	4%
Hookworm	6	4	67%	5	83%	5	83%	2	33%	6	100%	5	83%	1	17%	4	67%	2	33%	0	0%
*Opisthorchis*	5	2	40%	5	100%	3	60%	3	60%	3	60%	2	40%	3	60%	2	40%	0	0%	0	0%
*Strongyloides*	3	1	33%	3	100%	2	67%	1	33%	2	67%	2	67%	2	67%	1	33%	0	0%	0	0%
*Cryptosporidium*	5	3	60%	2	40%	3	60%	3	60%	4	80%	3	60%	3	60%	2	40%	1	20%	0	0%
*Taenia*	1	0	0%	1	100%	1	100%	1	100%	1	100%	0	0%	0	0%	1	100%	0	0%	0	0%
VIRUSES																					
Norovirus	37	14	38%	17	46%	18	49%	19	51%	15	41%	15	41%	17	46%	22	59%	8	22%	1	3%
Sapovirus	30	16	53%	14	47%	14	47%	13	43%	9	30%	9	30%	13	43%	18	60%	4	13%	0	0%
Adenovirus	21	11	52%	9	43%	8	38%	13	62%	12	57%	11	52%	8	38%	14	67%	1	5%	0	0%
Astrovirus	6	0	0%	1	17%	3	50%	1	17%	3	50%	1	17%	3	50%	2	33%	1	17%	0	0%
Rotavirus	99	60	61%	50	51%	78	79%***	85	86%***	75	76%	75	76%***	77	78%***	33	33%	13	13%	1	1%

p-values calculated from logistic regression for the presence of a symptom for each pathogen, * *p* < 0.05, ** *p* < 0.01, *** *p* < 0.001

Viruses were highly prevalent in pediatric diarrhea cases, accounting for 46% of acute cases in subjects 5 years old or younger, compared to 4.8% of acute cases in adults. The most prevalent virus was rotavirus, which accounted for 24% of acute diarrhea cases in children aged 2–5 years, compared to 2% in adults. After rotavirus, the most prevalent viruses were norovirus (19%), sapovirus (16%), and adenovirus (11%). Unlike bacteria, which often showed comparable carriage rates between cases and controls, viral infections were highly associated with cases. Rotavirus was strongly associated with acute diarrhea cases (*p* < 10^− 6^) with an aOR of 8.2. Norovirus was associated with acute diarrhea cases (*p* < 0.01) with an aOR of 2.8, and adenovirus and sapovirus were also associated with diarrhea cases (both *p* < 0.05) with an aOR of 2.1 and 1.9, respectively.

### Pathogen-associated symptoms

Clinical signs and symptoms of fever, abdominal pain, nausea, vomiting, fatigue, anorexia, and stool characteristics presented with each diarrhea case are shown in Table 5. Overall, clinical presentation for acute diarrhea was largely non-specific with respect to enteric pathogens, with abdominal pain as the most common symptom reported among diarrheal cases, followed by nausea, fatigue, vomiting, and anorexia. We found statistically significant associations between being positive for *Shigella* and the presence of blood and mucous in stool (both *p* < 10^− 5^). Bloody stools were also found to be positively associated with ETEC and *Campylobacter*, although at low numbers. We found that *Giardia* infection was also associated with mucous in the stool (*p* < 0.05). Finally, we found that rotavirus was strongly associated with nausea (*p* < 0.001), vomiting (*p* < 10^− 5^), anorexia (*p* < 10^− 6^), and watery stool (*p* < 10^− 8^). Vomiting was commonly reported among cases with viral infections (68%) compared to bacterial infections (41%) and parasitic infections (51%). We also carried out logistic regression to see if the presence of a particular pathogen was positively associated with hospitalization (IPD cases). We found only two associations, *Shigella* was weakly associated with hospitalization (aOR 3.2, *p* < 0.05) and rotavirus was strongly associated with hospitalization (aOR 8.6, *p* < 10^− 7^) (data not shown), reflecting what is seen above with the association with more severe diarrhea symptoms of watery diarrhea, and blood or mucous in the stool.

### Pathogen prevalence at follow-up

**Fig. 2:**
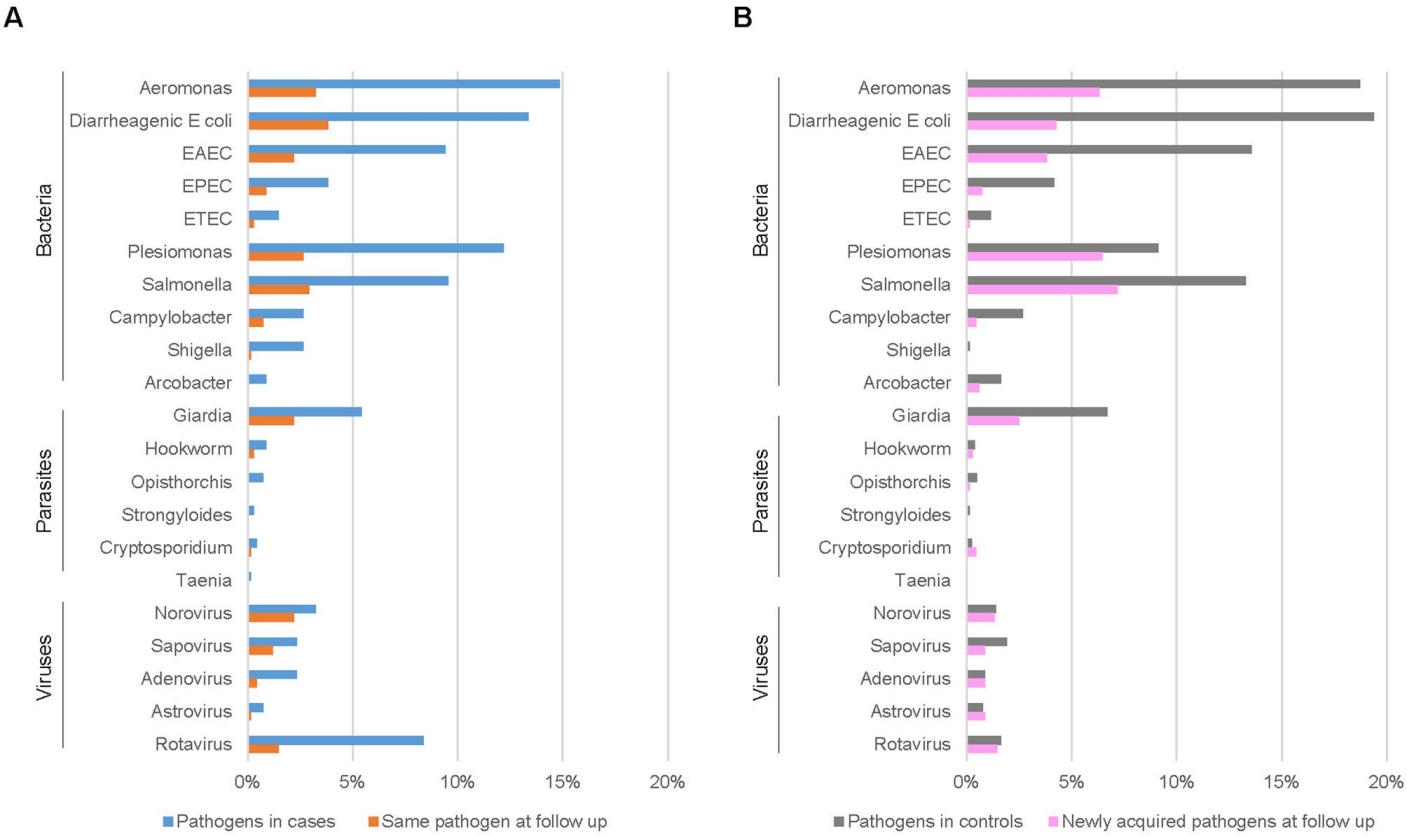
Enteropathogen prevalence at follow-up. (**A**) Pathogen prevalence in a subset of cases with follow-up data for (blue) and the prevalence for the same pathogen identified at enrollment and at the follow-up time point (orange). (**B**) Pathogen prevalence for asymptomatic control subjects (gray) and pathogen prevalence for newly acquired pathogens for follow-up cases (pink), defined as pathogens identified in the follow-up time point that were not identified in the enrollment time point

We conducted follow-up visits for 675 subjects who were cases at enrollment to determine what treatments were provided, whether the diarrhea had resolved, and to analyze pathogen prevalence in stool samples 7 to 21 days after enrollment. This enables us to determine whether any of the pathogens identified at enrollment were cleared following treatment or whether the subjects were still shedding that pathogen, as well as identify if/what new enteropathogens may have been acquired in that span of time, to provide some indication of endemicity. Overall, 92% of subjects at follow-up reported that their diarrhea had resolved. Figure 2A shows the pathogen prevalence at enrollment for the subset of cases that we had follow-up data on, as well as the prevalence of the same pathogen(s) in the follow-up visit, Fig. 2B. Overall, we found that there is substantially lower prevalence of enteropathogens across the board in follow-up subjects. For most bacterial pathogens, approximately 80% of the cases were cleared of the original bacterial enteropathogen by follow-up. For parasitic pathogens, *Giardia* and hookworm, approximately 60% of cases were cleared of the original parasitic infection. For viruses, the results were more varied. For rotavirus and adenovirus cases detected at enrollment, the viruses were still detected at follow-up in approximately 20% of these cases. By contrast, approximately 30–50% of norovirus and sapovirus cases were still positive at follow-up, suggesting that viral shedding for these two viruses may be longer.

In addition to looking at the prevalence of pathogens identified at enrollment, we also looked for the prevalence of newly acquired pathogens since enrollment to get an indication of the endemicity of many of these pathogens. Overall follow-up subjects had approximately 50% lower prevalence than controls at 7–14 days following treatment (Fig. 2B), suggesting (1) that treatment, typically through the use of antibiotics, does reduce the overall burden for bacterial and parasitic enteropathogens, at least temporarily, and (2) that even in the short time period of 7–21 days post-treatment, subjects were acquiring new enteropathogens and beginning to resemble the control population. By contrast, the prevalence of newly acquired viral enteropathogens in this short period was almost identical to the prevalence in controls, demonstrating the high level of endemicity of these viral pathogens in this population and environment.

### Antimicrobial sensitivity testing

**Fig. 3:**
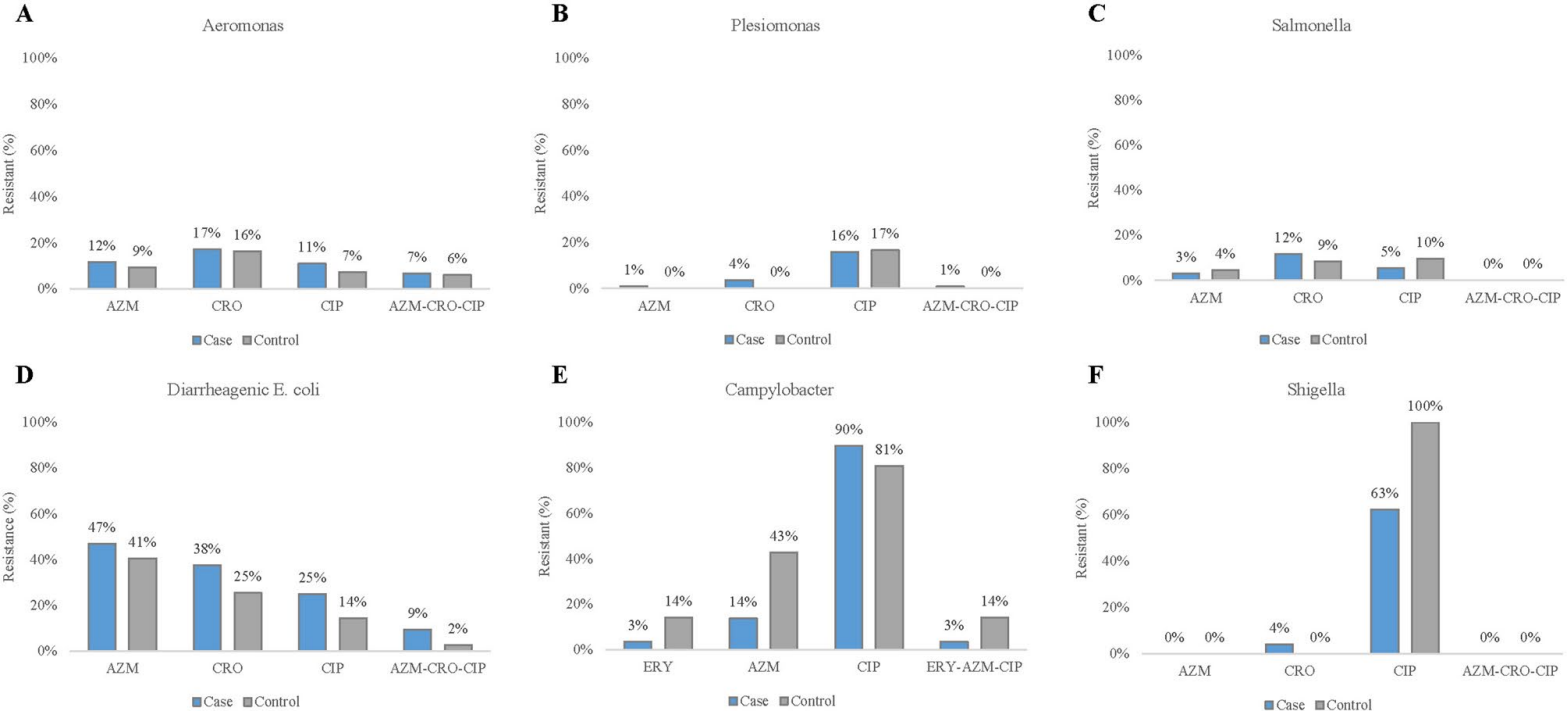
Antimicrobial resistance profile for selected enteric bacterial pathogens. Antimicrobial resistance profiles shown as a percentage for *Aeromonas* (**A**), *Plesiomonas* (**B**), *Salmonella* (**C**), diarrheagenic *E. coli* (**D**), *Campylobacter* (**E**), and *Shigella***(F)** isolates from cases (blue) and controls (gray) for the antibiotics azithromycin (AZM), ceftriaxone (CRO), ciprofloxacin (CIP), and erythromycin (ERY)

All bacterial isolates (607 isolates of cases, and 512 isolates of controls, Supplement Table 2) were tested for antimicrobial resistance. Antimicrobial resistance profiles of bacterial isolates of cases and controls are shown in Fig. 3. There was a variable resistance to the first line of antibiotic treatment, AZM-CRO-CIP, by different bacterial isolates with *Shigella* being notably resistance to CIP (Fig. 3F) and to SXT (21/25, 84%) (Supplement Table 6.1). *Aeromonas*,* Plesiomonas*,* Salmonella*, and DEC had low to moderate resistance rates to all three antibiotics (Fig. 3A-D). According to the intrinsic resistance of *Aeromonas* spp., all 431 *Aeromonas* isolates in this study were 100% resistant to AMP [30]. Notably, *Campylobacter* isolates isolated from control samples had high resistance rates to AZM and CIP, respectively (Fig. 3E). *C. coli* and *C. jejuni* isolates were highly resistant to CIP ([16/16, 100%] and [22/27, 82%]) and NAL ([16/16, 100%] and [23/27, 85%]), respectively ((Supplement Table 6.2). All *Arcobacter* isolates were resistance to AZM while 31-38% were resistant to CIP, ERY, and NAL (Supplement Table 6.2). A MDR pattern (resistant to AZM-CRO-CIP) was found in 21 *Aeromonas* isolates, 19 DEC isolates, and 1 *Plesiomonas* isolate in cases and controls.

## Discussion

Among the patients presented with acute diarrheal illness in this study, bacteria were the most common pathogen identified (42%), followed by viruses (21%), while parasites only accounted for a relatively small percentage of cases (8%), where a potential etiological agent could be identified. In general, we observed a relatively high carriage of enteropathogenic bacteria in both diarrheal cases and age-matched controls. Among bacterial enteropathogens, we found that *Shigella* spp., ETEC-ST, and *Plesiomonas* had a statistically significant association with acute diarrhea cases. We did find higher than expected prevalence of *Shigella* in children age 2–5 years old and adolescents (both at 7%), which could indicate a shift in the epidemiology of this pathogen in Cambodia from previous years [6]. We found a relatively low prevalence of parasitic enteropathogens, with *Giardia* being the most prevalent, particularly in children age 2–5 years old, where prevalence was 10% in acute cases and 11% overall. While lower than expected, its prevalence in this age group in both cases and controls is consistent with prior studies in Cambodia and Thailand [6, 31].

In follow up visits conducted 7 to 21 days after the initial visit, we found that 92% of subjects reported their acute diarrhea had resolved. In terms of shedding of bacterial pathogens, we found that there was an approximately 80% clearance rate of the initially identified bacterial pathogen, compared to a ~ 60% clearance rate for parasites (mainly *Giardia* and hookworm), and varying clearance rate for viral pathogens (~ 80% for rotavirus and adenovirus, 70% for sapovirus and 50% for norovirus). Although follow-up testing is relatively rare in clinical diarrhea studies, our results are consistent with prior reports of relatively short shedding duration of 9–12 days for bacterial enteropathogens such as STEC [32, 33], moderate shedding duration of 10–20 days for rotavirus [34], and long shedding duration of 14–56 days for norovirus [35–37]. We also observed a high rate of acquiring new enteropathogens at follow-up that were not identified in the initial visit. For bacterial and parasitic enteropathogens, the prevalence of newly acquired pathogens following treatment at follow-up was approximately 50% of the prevalence rate observed in asymptomatic controls; for viral pathogens, the prevalence of newly acquired viral pathogens in follow-ups was comparable to what was observed in controls. These findings highlight both the fact that treatment (largely antibiotic-based) was mostly successful at clearing the initially identified pathogen, but also that within a relatively short period of 7–21 days, subjects were acquiring new enteropathogens and well on their way to resembling the high carriage rates observed in the control population, underscoring the high endemicity of these pathogens in this population and/or environment.

The high carriage rate and lack of significant difference in prevalence for bacterial enteropathogens between cases and asymptomatic controls have been observed previously in Southeast Asia. Two case-control studies conducted in 2010 and 2016–2018 in Thailand reported high carriage rates of *Campylobacter*, *Plesiomonas*, *Salmonella*, and DEC [31, 38]. This has also been seen in other LMICs both in Asia, such as Nepal [39], and outside of Asia such as in the Central African Republic [40] and Columbia [41], albeit with a different pathogen prevalence profile and is reflected in the generally low attributable fractions for these pathogens in the Global Enteric Multicenter Study [42]. Case-control studies of diarrhea in high-income countries are less common, but interestingly, a study in Denmark found very low carriage rates of bacterial enteropathogens in controls, indicating that endemicity and environmental factors may contribute to carriage rate [43]. The significance of a high rate of bacterial carriage in age-matched controls suggests that other host factors may contribute to the conversion from asymptomatic carriage to the presentation of acute diarrhea. Another possibility is that the similarity between diarrhea cases and asymptomatic controls could be due to the detection of carriage pathogens among the study participants in both groups, rather than the detection of the true causative agents of acute diarrhea.

In this study we found that viral infections were highly prevalent in children, accounting for 29% of diarrhea cases in infants and 56% of cases in children 5 years old and younger, compared to 10% of cases in children and adolescents (6–17 years) and only 4% of cases in adults. The high frequency of rotavirus (31%) in young children in this study (2–5 years), is in line with a previous surveillance study in Cambodia collected from 2010 to 2016 which found that 50% of hospitalizations due to acute gastroenteritis in children under 5 years old is due to rotavirus [44], and another study in 2011 found that rotavirus was responsible for diarrhea in 26% of cases with children [14]. Our findings in this study period from 2020 to 2023, show that the disease burden of rotavirus in Cambodia remains high and underscores the importance of initiatives to implement a nationwide childhood rotavirus vaccination program [45].

Our finding of norovirus prevalence of 9% in infants and 7% in children aged 2–5 years is similar to those observed in previous studies in Cambodia, both in pediatric population in 2004 to 2006 where 7% positive rate was reported [14, 46] and in a mixed population from 2012 to 2018, where 9% positivity rate was obtained [6], suggesting that after rotavirus, norovirus remains the most common viral enteropathogen in Cambodia. Norovirus GII accounted for 91% of norovirus cases in this study. Further research is needed to determine if there has been a shift in the GII genotypes as norovirus outbreaks have emerged in East and Southeast Asia with a highly infectious strain GII.2 [P16] genotype, which was recently reported in Malaysia, Japan, and Taiwan between 2014 and 2018 [47–49]. There was also an outbreak caused by norovirus GII.3[P25] in Thailand in early 2023 [50] whereas the predominant strain was GII.3[P12] in Thailand between 2019 and 2020 [51], potentially suggesting norovirus as a re-emerging pathogen. For adenovirus, our incidence rate of 4.5% and 6.6% for infants and children is also in line with the previous study in a pediatric population in Cambodia which showed an incidence rate of 4.4% [14]. Finally, sapovirus, a relatively new viral enteropathogen had a prevalence of 10.0% among children age 2–5 years with diarrhea, which is among the highest rates reported so far in literature for this region, predominantly made up of the Sapo-124 genogroups. Prior studies in Thailand have reported sapovirus rates of 3.4% [52], and a recent study in China reported 0.5% [53], but this virus is often not tested for in diarrhea surveillance studies and our findings suggest it may be on the rise as a viral enteropathogen in the pediatric population in Southeast Asia.

When evaluating symptoms based on etiology, abdominal pain was the most common symptom among cases with bacterial and parasitic etiology, as compared to vomiting in cases with a viral etiology and that vomiting combined with abdominal pain showed high likelihood of a viral etiology. We found several significant associations between clinical symptoms and an enteropathogen. *Shigella* was associated with blood and mucous in the stool which is consistent with prior studies on the clinical presentation of shigellosis [54]. *Giardia* was associated with mucous in the stool. Rotavirus was associated with nausea, vomiting, anorexia, and watery stool. We found that fever was not a good differentiating indicator between bacterial, parasitic, and viral etiologies, however, fever has been reported to be common in rotavirus [55], norovirus [27], nontyphoidal *Salmonella* spp [55] and *Campylobacter* [27, 55, 56] infections.

Approximately 56% of cases that were treated with antibiotics were not infected by enteropathogenic bacteria that could be identified by stool culture, indicating a high rate of antibiotic overuse. AZM was recently recommended as the first-line antibiotic for the treatment of acute watery, febrile diarrhea, and dysentery [57] and our results suggest it is still an effective antimicrobial with the exception of DEC as their resistance rates were high. An overall low resistance to the first line of treatment (AZM-CRO-CIP) by studied isolates suggests that these antibiotics should remain as the treatment of choice. For *Arcobacter* and *Campylobacter*, resistance to AZM and CIP was high among either case or control or both indicating that other drugs of choice, such as ERY should be considered [58, 59]. For Western travelers, AZM and CIP are mostly frequently prescribed by traveler’s clinics prior to travel for use in case of travelers’ diarrhea [60]. This study finds that DEC is significantly resistant to AZM/CIP, and *Shigella* and *Campylobacter* are significantly resistant to CIP, suggesting limitations on the use of these drugs when traveling to Southeast Asia.

There were some limitations to the present study. First, given the high carriage rate of many of the bacterial pathogens in asymptomatic controls, it is possible that in many acute diarrhea cases, the diarrhea was not caused by the bacterial enteropathogen we identified in the stool, but rather was a result of another pathogen not detected in our analysis or to host factors not captured by this study. Second, we carried out limited pathotyping or genetic characterization of these pathogens that might have better explained the etiology of acute diarrhea in this population. For example, there were equal carriage rates of DEC between cases and controls, but ETEC expressing heat-stable toxin was strongly associated with acute diarrhea but was rarely found in control cases.

## Conclusions

In this case-control study of acute diarrheal infection in Cambodia, we found that bacterial enteropathogens were the most prevalent etiological agent, followed by viral and parasitic pathogens. We found high carriage rates of bacterial pathogens such as DEC in asymptomatic control subjects, suggesting that there may be other contributing factors to acute diarrhea. We found strong associations with acute diarrhea for a subset of bacterial pathogens, such as ETEC and *Shigella* as well as for most of the viral pathogens tested, including rotavirus, norovirus, and sapovirus. Rates of rotavirus in children were particularly high, where it accounted for 31% of acute diarrhea cases in children age 2–5 years old, underscoring the urgent need for progress on rotavirus vaccination efforts in the region. Finally, we found substantial evidence of antimicrobial resistance in bacterial enteropathogens studied here, particularly AZM and CIP resistance in *E. coli* and CIP resistance in *Shigella* and *Campylobacter* spp. Our findings underscore the need for ongoing disease surveillance and monitoring of acute diarrhea in Southeast Asia and highlight the evolving etiologies and bacterial antimicrobial resistance patterns in the region over time.

## Electronic supplementary material

Below is the link to the electronic supplementary material.

Supplementary Material 1

## Data Availability

No datasets were generated or analysed during the current study.

## Declarations

### Disclaimers

Material has been reviewed by the Walter Reed Army Institute of Research. There is no objection to its presentation and/or publication. The opinions or assertions contained herein are the private views of the authors and are not to be construed as official or as reflecting true views of the Department of the Army or the Department of Defense. The investigators have adhered to the policies for protection of human subjects as prescribed in AR 70 to 25.
