# Supplementary material for: Understanding the etiology of diarrheal illness in Cambodia in a case-control study from 2020 to 2023
Source: Gut Pathog. 2025 May 22;17:32. doi: 10.1186/s13099-025-00709-0 (PMC12100842; doi:10.1186/s13099-025-00709-0)
Supplement: Supplementary file 1 — Supplementary Material 1 [file 13099_2025_709_MOESM1_ESM.pdf]

Supplemental Material

Supplemental Table 1: Primer sequences for DEC pathotype assays

DEC	Target gene(s)	Name	Sequence (5' to 3')	Reference
EAEC	*aaiC*	EAEC215F[1]	ATTGTCCTCAGGCATTTCAC	Boisen *et al.,* 2008
		EAEC215R[1]	ACGACACCCCTGATAAACAA	
EAEC	*aatA*	EAEC650F[1]	CTGGCGAAAGACTGTATCAT	Schmidt *et al.,* 1995
		EAEC650R[1]	CAATGTATAGAAATCCGCTGTT	
EIEC	*ipaH*	EIEC423F[3]	CTGGTAGGTATGGTGAGG	Luscher and Altwegg, 1994
		EIEC423R[3]	GGAGGCCAACAATTATTTCC	
EPEC/ EHEC	*eae*	EPEC881F[1]	CCCGAATTCGGCACAAGCATAAGC	Luscher and Altwegg, 1994
		EPEC881R[1]	CCCGGATCCGTCTCGCCAGTATTCG	
EPEC	*bfpA*	EPEC300F[1]	GGAAGTCAAATTCATGGGGG	Taniuchi *et al.,* 2012
		EPEC300R[1]	GGAATCAGACGCAGACTGGT	
ETEC	*LT*	ETEC508F[2]	CACACGGAGCTCCTCAGTC	Taniuchi *et al.,* 2012
		ETEC508R[2]	CCCCCAGCCTAGCTTAGTTT	
	STp	STp_VX67356F[2]	TCTTTCCCCTCTTTTAGTCAG	Rodas, *et al.,* 2009
		STp_VX67356R[2]	ACAGGCAGGATTACAACAAAG	
	STh	STh_ST64111F[2]	TTCACCTTTCCCTCAGGATG	
		STh_ST64111R[2]	CTATTCATGCTTTCAGGACCA	

*Table modified from Taniuchi, 2012.[1,2, and 3] indicate the multiplex set.

The multiplex PCR reactions contain 12.5µL of 2X KAPA Taq Ready Mix with Dye, 3µL of DNA template, 4 µL of primer master mix, and 5.5µL of sterile deionized water to a final volume of 25µL. The thermal cycling profile is as follows: 94°C for 3 min; followed by 26 cycles of 94°C for 1 min, 55°C for 1.5 min, and 72°C for 1.5 min, with a final extension at 72°C for 10 min.

Supplemental Table 2: Details of antibiotics used for antimicrobial susceptibility test on all bacterial isolates

Pathogens	No. of Isolates		Antibiotics						
	Case	Control	AMP	AZM	CIP	CRO	ERY	NAL	SXT
Aeromonas spp.	180	148	✓	✓	✓	✓	-	✓	✓
Arcobacter spp.	11	13	-	✓	✓	-	✓	✓	-
Campylobacter spp.	29	21	-	✓	✓	-	✓	✓	-
Diarrheagenic *E. coli*	160	154	✓	✓	✓	✓	-	✓	✓
Plesiomonas shigelloides	107	71	✓	✓	✓	✓	-	✓	✓
Salmonella spp.	93	104	✓	✓	✓	✓	-	✓	✓
Shigella spp.	24	1	✓	✓	✓	✓	-	✓	✓
Vibrio parahaemolyticus	2	0	✓	✓	✓	✓	-	✓	✓
Yersinia enterocolitica	1	0	✓	✓	✓	✓	-	✓	✓

Antibiotics: ampicillin (AMP), azithromycin (AZM), ciprofloxacin (CIP), ceftriaxone (CRO), erythromycin (ERY), nalidixic acid (NAL), and trimethoprim/sulfamethoxazole (SXT).

Supplemental Table 3: Distribution of subject enrollment across the enrolling hospitals

Study site	No. of subjects			
	**Cases**	**Controls**	**Follow up cases**	**Grand Total**
Anlong Veng Referral Hospital	254	196	146	596
Battambang Referral Hospital	41	42	9	92
Military Reginal 5 Hospital	198	184	145	527
Oudormeanchey Hospital	298	286	274	858
SvayPor Health Center	127	83	101	311
**Grand Total**	**918**	**791**	**675**	**2384**

Supplemental Table 4: Details of infection types in cases

**Infection types**	**Cases (N=918)**	
	**n**	**%**
Bacteria (sole)	198	22%
Bacteria and Parasite	29	3%
Bacteria more than 1 species	102	11%
Parasite (sole)	23	3%
Parasites more than 1 species	1	0%
Virus (sole)	110	12%
Virus and Bacteria	51	6%
Virus and Parasite	11	1%
Virus more than 1 species	5	1%
Virus and Bacteria and Parasite	3	0%
**Grand Total**	**533**	**58%**

Supplemental Table 5: Details of enteropathogen subtypes

Organism	Subtype	% of organism	CASE		CONTROL	
			N	%	N	%
Aeromonas	*Aeromonas caviae*	41.2%	81	8.8%	54	6.8%
	Aeromonas hydrophila	23.5%	38	4.1%	39	4.9%
	Aeromonas veronii biovar sobria	35.4%	61	6.6%	55	7.0%
Arcobacter	*Arcobacter butzleri*	66.7%	11	1.2%	5	0.6%
	Arcobacter cryaerophilus	8.3%	0	0.0%	2	0.3%
	Arcobacter spp.	25.0%	0	0.0%	6	0.8%
Campylobacter	*Campylobacter coli*	32.0%	11	1.2%	5	0.6%
	Campylobacter hyointestinalis	8.0%	0	0.0%	4	0.5%
	Campylobacter jejuni subsp. doylei	2.0%	1	0.1%	0	0.0%
	Campylobacter jejuni subsp. jejuni	52.0%	17	1.9%	9	1.1%
	Campylobacter spp.	6.0%	0	0.0%	3	0.4%
Diarrheagenic *E. coli*	EAEC-aaiC	23.6%	38	4.1%	36	4.6%
	EAEC-aaiC, aatA	20.1%	27	2.9%	36	4.6%
	EAEC-aatA	22.3%	35	3.8%	35	4.4%
	EIEC-ipaH	1.3%	3	0.3%	1	0.1%
	EPEC-eae	21.7%	35	3.8%	33	4.2%
	EPEC-eae, bfpA	0.0%	0	0.0%	0	0.0%
	ETEC-lt	3.8%	6	0.7%	6	0.8%
	ETEC-lt, st-Ia	1.9%	4	0.4%	2	0.3%
	ETEC-lt, st-Ib	0.3%	1	0.1%	0	0.0%
	ETEC-st-Ia	1.9%	4	0.4%	2	0.3%
	ETEC-st-Ib	1.9%	6	0.7%	0	0.0%
	STEC-stxII (LF)	1.0%	1	0.1%	2	0.3%
	STEC-stxII, eae (LF)	0.3%	0	0.0%	1	0.1%
Plesiomonas	*Plesiomonas shigelloides*	100.0%	107	11.7%	71	9.0%
Salmonella	*Salmonella* group B	23.4%	26	2.8%	20	2.5%
	Salmonella group C	39.1%	34	3.7%	43	5.4%
	Salmonella group D	5.1%	4	0.4%	6	0.8%
	Salmonella group E	18.3%	18	2.0%	18	2.3%
	Salmonella group F	0.0%	0	0.0%	0	0.0%
	Salmonella group G	5.6%	4	0.4%	7	0.9%
	Salmonella group H	1.0%	1	0.1%	1	0.1%
	Salmonella group I	3.0%	4	0.4%	2	0.3%
	Salmonella group K	0.5%	0	0.0%	1	0.1%
	Salmonella group N	0.0%	0	0.0%	0	0.0%
	Salmonella group O	3.0%	1	0.1%	5	0.6%
	Salmonella spp.	1.0%	1	0.1%	1	0.1%

Supplemental Table 5: Details of enteropathogen subtypes (Continued)

Organism	Subtype	% of organism	CASE		CONTROL	
			N	%	N	%
Shigella	*Shigella boydii* 2	8.0%	2	0.2%	0	0.0%
	Shigella flexneri 1a	8.0%	2	0.2%	0	0.0%
	Shigella flexneri 2a	28.0%	7	0.8%	0	0.0%
	Shigella flexneri 4a variant	24.0%	5	0.5%	1	0.1%
	Shigella sonnei	32.0%	8	0.9%	0	0.0%
Vibrio	*Vibrio parahaemolyticus*	100.0%	2	0.2%	0	0.0%
Yersinia	*Yersinia enterocolitica*	100.0%	1	0.1%	0	0.0%
Norovirus	NoV-GI	14.6%	5	0.5%	2	0.3%
	Nov-GII	85.4%	32	3.5%	9	1.1%
Sapovirus	Sapo-124	78.3%	25	2.7%	11	1.4%
	Sapo-5	21.7%	5	0.5%	5	0.6%

Supplemental Table 6.1: Details of antimicrobial resistance profiles for enteric bacterial pathogens

Pathogens	AMP				AZM				CIP				CRO				NAL				SXT			
	CASE	%	CONTROL	%	CASE	%	CONTROL	%	CASE	%	CONTROL	%	CASE	%	CONTROL	%	CASE	%	CONTROL	%	CASE	%	CONTROL	%
Aeromonas	180	100%	148	100%	21	12%	14	9%	20	11%	11	7%	31	17%	24	16%	99	55%	87	59%	48	27%	31	21%
**Diarrheagenic *E. coli***	128	80%	110	71%	75	47%	62	40%	40	25%	22	14%	60	38%	39	25%	99	62%	78	51%	93	58%	66	43%
Plesiomonas	52	49%	38	54%	1	1%	0	0%	17	16%	12	17%	4	4%	0	0%	22	21%	14	20%	12	11%	7	10%
Salmonella	42	45%	48	46%	3	3%	5	5%	5	5%	10	10%	11	12%	9	9%	18	19%	26	25%	19	20%	32	31%
Shigella	15	63%	1	100%	0	0%	0	0%	15	63%	1	100%	1	4%	0	0%	17	71%	0	0%	20	83%	1	100%
Vibrio	2	100%	NI	NI	0	0%	NI	NI	0	0%	NI	NI	0	0%	NI	NI	0	0%	NI	NI	0	0%	NI	NI
Yersinia enterocolitica	1	100%	NI	NI	0	0%	NI	NI	0	0%	NI	NI	0	0%	NI	NI	0	0%	NI	NI	0	0%	NI	NI

Antimicrobial resistance profiles are shown as a percentage for selected enteric bacterial pathogens from cases and controls for the antibiotics ampicillin (AMP), azithromycin (AZM), ciprofloxacin (CIP), ceftriaxone (CRO), nalidixic acid (NAL), and trimethoprim/sulfamethoxazole (SXT). NI - no isolate.

Supplemental Table 6.2: Details of antimicrobial resistance profiles for enteric bacterial pathogens (*Arcobacter* and *Campylobacter*)

Pathogens	AZM				CIP				ERY				NAL			
	CASE	%	CONTROL	%	CASE	%	CONTROL	%	CASE	%	CONTROL	%	CASE	%	CONTROL	%
Arcobacter	11	100%	13	100%	1	9%	8	62%	4	36%	5	38%	2	18%	8	62%
Campylobacter	4	14%	9	43%	26	90%	17	81%	1	3%	3	14%	27	93%	18	86%

Antimicrobial resistance profiles are shown as a percentage for selected enteric bacterial pathogens from cases and controls for the antibiotics azithromycin (AZM), ciprofloxacin (CIP), erythromycin (ERY), and nalidixic acid (NAL).
